# Associations between childhood adverse experiences and medication adherence in asthma patients: a dual variable-centered and person-centered analysis of attachment security and mentalization

**DOI:** 10.3389/fpsyt.2026.1754985

**Published:** 2026-07-29

**Authors:** Fang Wang, Yun Qian, Limin Tian, Yaoyao Cui

**Affiliations:** Department of Respiratory and Critical Care Medicine, Beijing Genertec Aerospace Hospital, Beijing, China

**Keywords:** asthma patients, attachment security, childhood adverse experiences, medication adherence, mentalization

## Abstract

**Background:**

Childhood adverse experiences are increasingly recognized as risk factors for poor health outcomes in chronic diseases. However, their influence on medication adherence in asthma patients remains underexplored, with limited research addressing the key psychological constructs of attachment security and mentalization. This study adopts both variable-centered and person-centered perspectives to investigate the association between childhood adverse experiences and medication adherence among asthma patients, as well as the heterogeneity within this relationship.

**Methods:**

A cross-sectional design with convenience sampling was employed. From July to August 2025, 525 asthma patients were recruited from four tertiary hospitals in Beijing, China. Participants completed validated scales measuring childhood adverse experiences, attachment security, mentalization, medication adherence, and relevant demographic information. Structural equation modeling was used to examine the indirect statistical associations of attachment security and mentalization. Latent profile analysis identified subgroups based on attachment security and mentalization profiles.

**Results:**

Findings demonstrated a significant negative correlation between childhood adverse experiences and medication adherence in asthma patients. From the variable-centered perspective, higher levels of childhood adverse experiences were associated with lower attachment security, which was in turn associated with lower mentalization capacity; this sequence showed a significant indirect association with medication adherence. The person-centered analysis revealed three latent profiles characterized by “high attachment security–high mentalization,”(7.6%) “moderate attachment security–moderate mentalization,”(83.9%) and “low attachment security–low mentalization.”(8.5%).

**Conclusion:**

This study identified significant direct and indirect statistical associations among childhood adverse experiences, attachment security, mentalization, and medication adherence among asthma patients. Attachment security and mentalization statistically mediated the relationship between childhood adverse experiences and medication adherence within a theoretically specified model. A person-centered approach revealed heterogeneous subgroups, underscoring the need for tailored interventions. Clinically, integrating trauma-informed psychological screening may enhance adherence and improve asthma management outcomes.

## 1 Introduction

Asthma is a highly heterogeneous chronic inflammatory respiratory disease characterized by protracted course (1), frequent exacerbations, environmental sensitivity, and the need for long-term, regular medication to maintain disease control (2, 3). Despite significant advances in asthma pharmacotherapy and management guidelines (4, 5), a substantial proportion of asthma patients worldwide experience poor disease control (6). This is largely attributed not to inadequate healthcare access but to persistently low medication adherence (7). Traditional research has primarily focused on external factors such as medication availability, patient education, and healthcare resources to explain poor adherence (8, 9). However, recent evidence suggests that early psychosocial experiences can profoundly shape individuals’ emotional regulation, health beliefs, and decision-making behaviors, thereby exerting a deep impact on chronic disease management (10, 11). Notably, childhood adverse experiences may be associated with patients’ understanding, trust, and commitment to long-term treatment through their links with emotional regulation strategies, self-efficacy, and basic expectations regarding others’ reliability (12–14). Hence, investigating how childhood adverse experiences are associated with asthma patients’ disease management behaviors in adulthood is of great theoretical significance for elucidating the underlying psychological mechanisms contributing to asthma control difficulties. Nevertheless, existing studies have yet to systematically map this causal chain, and the core psychological mechanisms remain inadequately clarified. This study aims to fill this critical gap.

Childhood adverse experiences refer to cumulative adversities experienced early in life, including emotional neglect, physical abuse, and family dysfunction (15). Such stressors can alter brain structure and stress response patterns during sensitive neurodevelopmental periods through chronic activation of the stress system, thereby impacting adult emotional regulation, stress perception, trust levels, and health behavior patterns (16, 17). Previous research has linked childhood adverse experiences to increased risks of somatic diseases, impaired health decision-making, and treatment avoidance in adulthood (18, 19). However, these studies have predominantly examined disease risk (20), with fewer focusing on chronic disease management behaviors such as medication adherence. Effective asthma management requires patients to possess emotional regulation skills, trust in healthcare systems, and motivation to adhere to treatment plans (21, 22). Childhood adverse experiences may undermine these psychological capacities, decreasing adherence. Therefore, this study addresses two key questions: (1) Are childhood adverse experiences negatively associated with medication adherence in asthma patients? (2) What are the internal psychological mechanisms linking childhood adverse experiences to adherence?

Attachment theory posits that early interactions with caregivers shape individuals’ trust in others, self-concept, and stress coping strategies, serving as foundational drivers of adult psychological and behavioral patterns (23, 24). Attachment security is considered a relational and emotional regulation resource associated with more stable health behaviors in the face of chronic treatment demands like asthma medication management (25). In contrast, insecure attachment is associated with avoidance, anxiety, or low expectations of others’ reliability, which may weaken trust in medical advice and reduce persistence with long-term treatment (26). Attachment security also influences physiological stress systems, such as the hypothalamic-pituitary-adrenal (HPA) axis, potentially impacting chronic inflammatory disease outcomes (27, 28). Prior studies have shown attachment patterns predict adherence differences in chronic diseases like diabetes and hypertension (29, 30); however, research on asthma remains limited. Notably, the mediating role of attachment security between childhood adverse experiences and asthma medication adherence, as well as cultural variations in this relationship, have yet to be fully explored. Incorporating attachment security into the explanatory framework promises to deepen understanding of asthma behavior management and expand attachment theory in health behavior research.

Mentalization—the capacity to understand one’s own and others’ mental states, including emotion recognition, intention inference, self-reflection, and emotion regulation (31, 32)—is considered a core mechanism linking early relational experiences to adult psychological adaptation (33). Patients with robust mentalization skills better discern the relationship between bodily symptoms and emotional responses, avoiding maladaptive medication use driven by anxiety or catastrophizing (34, 35). They also comprehend medical advice realistically, promoting consistent medication routines. Conversely, impaired mentalization leads to emotional dysregulation or excessive rationalization under stress, destabilizing health behaviors (36, 37). In asthma, emotional activation and cognitive biases often exaggerate symptoms, causing overuse of rescue medication or neglect of maintenance therapy (38).

However, to our knowledge, direct empirical studies explicitly examining mentalization in asthma or other chronic respiratory diseases remain very limited. Existing respiratory research has more commonly examined related but narrower constructs, such as alexithymia, emotional awareness, symptom perception, illness perception, and self-reflectiveness. For example, studies have shown that alexithymia is relatively common among patients with asthma and is associated with poorer quality of life, psychiatric comorbidity, impaired symptom recognition, and worse disease control. Other work has also examined alexithymia and self-reflectiveness in bronchial asthma, suggesting that difficulties in identifying and interpreting emotional states may be clinically relevant in patients with reduced respiratory functioning. Nevertheless, alexithymia and self-reflectiveness do not fully capture mentalization, which refers to a broader capacity to understand both one’s own and others’ mental states in interpersonal and health-related contexts. Therefore, the role of mentalization in asthma medication adherence, particularly within a developmental pathway linking childhood adverse experiences and attachment security, remains insufficiently clarified.

Based on these theoretical considerations, this study hypothesizes that childhood adverse experiences, attachment security, and mentalization may constitute a theoretically meaningful psychological pathway associated with medication adherence in asthma patients. To comprehensively test this, both variable-centered and person-centered approaches are required. Variable-centered methods reveal overall associations and mediation effects, aiding in generalizable theoretical model development (39). Person-centered methods identify heterogeneity in attachment security and mentalization profiles, uncovering behavioral mechanism variations across risk configurations (40). This dual approach is especially valuable for asthma patients, given their variable disease course and complex psychological states, which may produce distinct adherence patterns. Relying on a single method risks overlooking key subgroup differences.

Though prior research has identified childhood adverse experiences’ impact on asthma control and psychological function, systematic, continuous theoretical and empirical investigation of childhood adverse experiences’ influence on medication adherence via attachment security and mentalization remains lacking. Moreover, most studies focus on Western populations, neglecting cultural influences on attachment, emotional expression, and treatment behaviors, underscoring the importance of exploring these mechanisms within the Chinese context. This issue is particularly relevant in the Chinese context, where family interdependence, filial responsibility, and relational harmony are culturally emphasized. On the one hand, close family ties may provide emotional security, practical support, and medication reminders, thereby strengthening adherence-related behaviors. On the other hand, when early family environments are characterized by neglect, conflict, or inconsistency, the centrality of family relationships may intensify the long-term impact of childhood adversity on interpersonal trust and attachment security. In addition, Chinese cultural norms often encourage emotional restraint and indirect emotional expression to maintain interpersonal harmony. Such norms may enhance sensitivity to contextual and relational cues, but they may also make it more difficult for some individuals to explicitly identify, verbalize, and regulate their own emotional states. In patients with asthma, this may be especially important because poor differentiation between emotional distress and respiratory symptoms can contribute to maladaptive illness interpretations and inconsistent medication use. Therefore, examining attachment security and mentalization in Chinese asthma patients may provide culturally informative insights into the psychological mechanisms underlying medication adherence.

**Figure 1:**
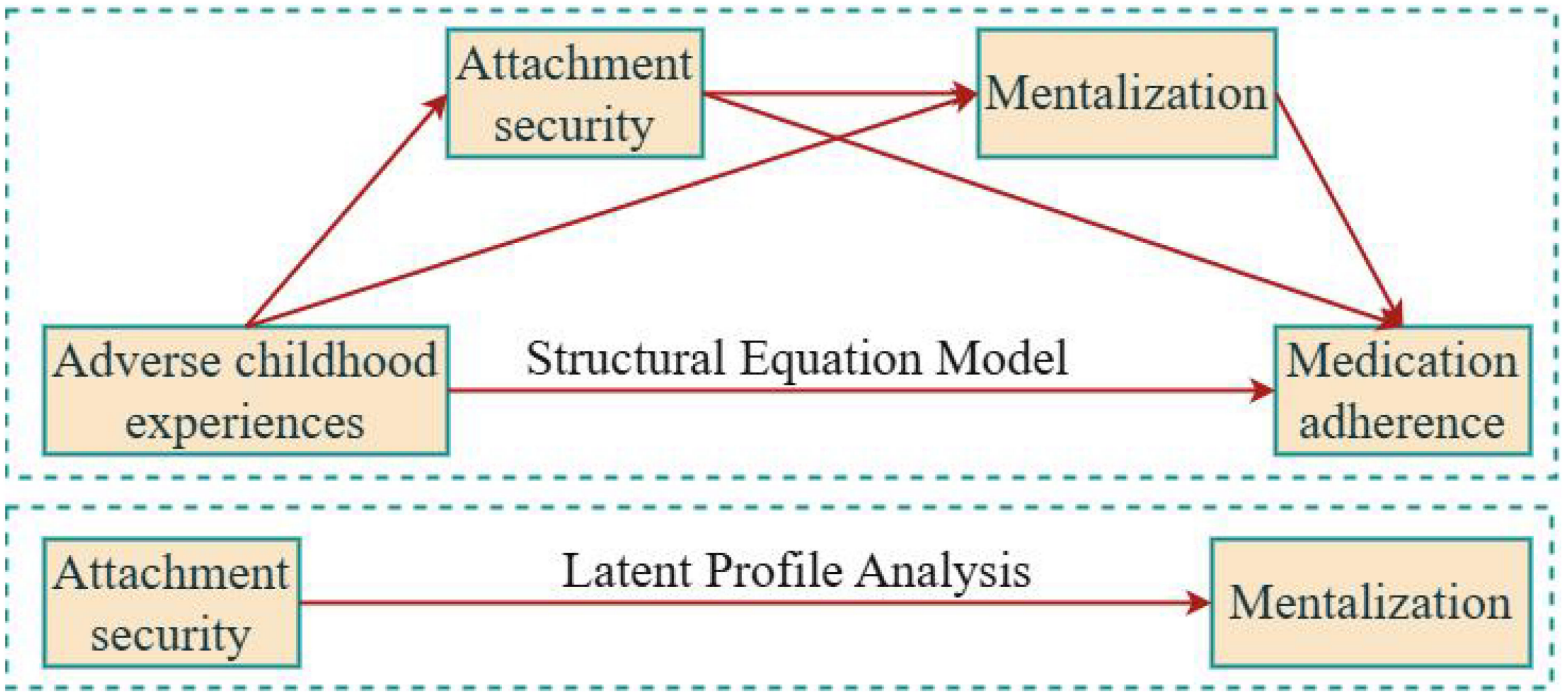
Research framework.

Therefore, this study employs a combined variable-centered and person-centered analytical strategy to systematically examine: (1) the direct effect of childhood adverse experiences on medication adherence; (2) the mediating roles of attachment security and mentalization; and (3) behavioral differences among psychological risk subtypes. The results will advance developmental psychopathology and chronic disease behavior management theories and provide clinical guidance for psychological screening, attachment and mentalization enhancement, and personalized adherence interventions in high-risk asthma patients, promoting an integrated medical-psychobehavioral management model. The research framework is shown in **Figure 1**.

## 2 Methods

### 2.1 Study design

A cross-sectional survey design was adopted to systematically assess the associations among childhood adverse experiences, attachment security, and mentalization on medication adherence in asthma patients, integrating both variable-centered and person-centered perspectives. Variable-centered analyses employed structural equation modeling (SEM) to test the serial mediation path: childhood adverse experiences → attachment security → mentalization → medication adherence. Person-centered analysis used latent profile analysis (LPA) to identify latent subgroups based on attachment security and mentalization profiles and compared adherence differences across these subgroups. Validated Chinese-language scales were used, and participants were recruited offline from outpatient clinics. Data collection was anonymous, using both electronic and paper questionnaires.

### 2.2 Ethical considerations

The study was reviewed and approved by the Academic Ethics Committee of Beijing Genertec Aerospace Hospital (No.:2025-1003-01). Participants provided informed consent online after reading study information detailing objectives, data confidentiality, and withdrawal rights. Data collection was anonymous, with strict confidentiality maintained and no data disclosed to third parties. Given the sensitive nature of childhood adverse experiences, participants were informed they could skip any items or withdraw at any time. Psychological support resources were provided to minimize potential distress.

### 2.3 Participants

#### 2.3.1 Recruitment

Participants were adult asthma patients diagnosed by hospital respiratory departments. Data collection occurred from July to August 2025 at inpatient and outpatient departments of three tertiary hospitals in Beijing. Researchers contacted respiratory department heads to explain the study purpose, methods, and risks and obtained their consent. Recruitment materials were distributed by medical staff to patients, who completed electronic or paper questionnaires.

#### 2.3.2 Inclusion and exclusion criteria

##### 2.3.2.1 Inclusion criteria

(1) age ≥ 18 years; (2) clinically diagnosed asthma per GINA guidelines (41); (3) current treatment with inhaled corticosteroids or ICS/LABA regimens; (4) cognitive ability sufficient to understand and complete questionnaires (MMSE ≥ 23) (42); (5) signed informed consent.

##### 2.3.2.2 Exclusion criteria

(1) comorbid severe psychiatric disorders (e.g., schizophrenia, bipolar disorder) affecting questionnaire validity; (2) severe organic diseases interfering with assessments; (3) questionnaires showing uniform or patterned responses or incomplete data.

#### 2.3.3 Minimum sample size

Sample size was estimated using G*Power 3.1 for SEM and LPA. For medium effect size (f²=0.15), α=0.05, power=0.95, and 13 predictors, a minimum of 189 participants was required. For LPA with 3–5 profiles, Bootstrap Likelihood Ratio Tests indicated each profile should have 50–70 cases for model robustness, totaling at least 350 participants. Accounting for 20% attrition, the study planned to recruit at least 400 valid participants.

#### 2.3.4 Data cleaning and sample characteristics

A total of 556 questionnaires were distributed. Based on criteria, 16 participants under 18 years, 9 untreated patients, and 6 with patterned responses were excluded, resulting in 525 valid questionnaires (94.42% response rate). Among these, 204 were male (38.9%) and 321 female (61.1%). Education levels concentrated at junior and senior high school (N = 234, 44.6%). Most participants were married (N = 336, 64.0%) and urban residents (N = 330, 62.9%). Detailed demographic information is presented in **Table 1**.

**Table 1:** Demographic information of the patients with asthma.

Variables	Items	Number (N)	Frequency (%)
Gender	Female	321	61.1
	Male	204	38.9
Educational Background	Primary school and below	138	26.3
	Middle school - High school	234	44.6
	Bachelor's degree or above	153	29.1
Marital status	Married	336	64.0
	Unmarried	117	22.3
	Divorced	52	9.9
	Widowed	20	3.8
Place of residence	Urban	330	62.9
	Rural area	195	37.1
Monthly income level	≤3000¥	177	33.7
	3001-6000¥	193	36.8
	6001-9000¥	116	22.1
	9001¥ and above	39	7.4
Smoking	Yes.	301	57.3
	No.	224	42.7
Drinking alcohol	Yes.	273	52.0
	No.	252	48.0
Age	53.87 ± 16.708		

### 2.4 Measurement instruments

#### 2.4.1 Childhood adverse experiences scale

The measurement items for childhood adverse experiences were adapted from the scale developed by Hawes, Lechowicz (43), comprising 23 items in a unidimensional format. Zhao, Li (44) translated this scale into two culturally adapted and psychometrically validated Chinese versions from distinct perspectives: the “Parents: self-report” and “Children: parent-report” versions, each containing 21 items. This study employed Zhao, Li (44) “Parents: self-report” version to assess childhood adverse experiences among asthma patients using a cumulative scoring method to evaluate overall exposure. An example item is: “Have you been seriously ill or injured or been in a serious accident?” Responses were rated on a 5-point Likert scale ranging from 1 = strongly disagree to 5 = strongly agree, with higher scores indicating more severe childhood adverse experiences. The Cronbach’s alpha for this scale in the current study was 0.824, demonstrating good internal consistency. Confirmatory factor analysis (CFA) confirmed excellent model fit, as shown in **Table 2**.

**Table 2:** Model fit indices of the core research variables.

Variables	GFI	AGFI	CFI	RMSEA	χ²/df	TLI	Cronbach’s α
Childhood adverse experiences	0.970	0.961	0.994	0.022	1.260	0.993	0.824
Attachment security	0.903	0.890	0.977	0.023	1.276	0.976	0.911
Mentalization	0.960	0.952	0.993	0.020	1.214	0.992	0.851
Medication adherence	0.952	0.929	0.958	0.066	3.296	0.948	0.902

#### 2.4.2 Medication adherence scale

The medication adherence scale was derived from the universal chronic disease medication adherence instrument developed by Naqvi, Hassali (45), consisting of 11 items covering domains such as “Non-adherence due to patient behavior” and “Cost-related non-adherence.” Wang, Wang (46) translated and validated the Chinese version, confirming its cultural appropriateness and reliability in chronic disease populations. This study used the scale to assess medication adherence in asthma patients, with items rated on a 5-point Likert scale (1 = strongly disagree, 5 = strongly agree). For example, one item asks, “Have you stopped medication because you are using other medications for different diseases?” Higher scores indicate better medication adherence. The scale demonstrated excellent internal consistency with a Cronbach’s alpha of 0.902 in this study. CFA results confirmed good model fit (see **Table 2**).

#### 2.4.3 Attachment security scale

Attachment security was measured using the Adult Attachment Scale developed by Fraley, Waller (47), which comprises two dimensions: anxiety (18 items) and avoidance (18 items). The anxiety dimension reflects concerns about the availability of others and fear of abandonment, while the avoidance dimension assesses comfort with intimacy, dependence, and emotional openness. This scale has been widely used in Chinese populations (48) and was translated and validated by Zhang, Hou (49), confirming its cultural validity and reliability. This study adapted the scale to assess attachment security in asthma patients, employing a 5-point Likert scale (1 = strongly disagree, 5 = strongly agree). For example, an item reads, “Do you agree that people around you love you very much?” Higher scores indicate greater attachment security. The Cronbach’s alpha was 0.911, indicating excellent internal consistency. CFA demonstrated good model fit (see **Table 2**).

#### 2.4.4 Mentalization scale

The mentalization scale used was developed by Dimitrijević, Hanak (50) to assess individuals’ mentalization capacity. It includes 28 items spanning three dimensions: self-related mentalization, other-related mentalization, and mentalization motivation. Wen, Fang (51) translated and validated the Chinese version among patient populations, demonstrating satisfactory cultural adaptation and reliability. This study employed this scale to evaluate mentalization in asthma patients, with responses rated on a 5-point Likert scale (1 = strongly disagree, 5 = strongly agree). An example item is: “Do you agree that when you feel uneasy, you are uncertain whether you are sad, afraid, or angry?” Higher scores indicate stronger mentalization ability. The scale showed good internal consistency with a Cronbach’s alpha of 0.851. CFA results confirmed good model fit as reported in **Table 2**.

### 2.5 Statistical analysis

Data analyses were conducted using Mplus 8, SPSS 27.0, and AMOS 30, with double data verification to ensure reproducibility. Statistical significance was set at p < 0.05, and effect sizes with 95% confidence intervals were reported. First, CFAs of individual variables were performed using AMOS 30 to assess model fit indices: GFI, AGFI, CFI, TLI ≥ 0.8; RMSEA ≤ 0.08. Concurrently, reliability analyses were performed in SPSS 27.0.

Second, independent samples t-tests examined demographic differences in medication adherence. Third, Harman’s single-factor test was conducted to assess common method bias. Fourth, descriptive statistics and correlation analyses were performed to report means, standard deviations, skewness, and kurtosis of study variables. Fifth, the PROCESS macro Model 6 in SPSS 27.0 was applied to test the serial mediation effect of attachment security and mentalization on the relationship between childhood adverse experiences and medication adherence.

Finally, latent profile models with 1 to 5 profiles were constructed using Mplus 8 to identify latent subgroups based on attachment security and mentalization. To reduce the risk of convergence on local maxima, each model was estimated using 500 random sets of starting values, with the 100 best solutions retained for final-stage optimization. For the bootstrap likelihood ratio test, 500 bootstrap draws were requested. Model selection was based on a combination of statistical fit indices and substantive interpretability, including the Akaike information criterion, Bayesian information criterion, sample-size adjusted Bayesian information criterion, entropy, Lo-Mendell-Rubin adjusted likelihood ratio test, bootstrap likelihood ratio test, average posterior classification probabilities, and the proportion of participants in each profile. The best loglikelihood value was checked for successful replication to support model stability. One-way ANOVA was then used to compare medication adherence across these latent classes.

## 3 Results

### 3.1 Common method bias analysis

Given the cross-sectional design of this study, participants may have been influenced by social desirability, consistency motives, or momentary emotions during questionnaire completion, potentially distorting their true responses. In addition, identical answering environments, fixed item order, and uniform scale formats may artificially inflate correlations among variables. To mitigate these risks, anonymity and data confidentiality were emphasized during data collection to reduce social desirability pressure. Reverse-scored items and neutral wording were also incorporated to further minimize common method bias.

To empirically assess common method variance, Harman’s single-factor test was conducted using exploratory factor analysis on all measurement items. The unrotated factor solution yielded 18 factors with eigenvalues greater than 1, and the first factor accounted for 13.933% of the variance, far below the 40% threshold. These results indicate that common method bias was unlikely to substantially influence the findings.

### 3.2 Demographic characteristics and medication adherence

Independent-sample t-tests were conducted to examine differences in medication adherence across sex, place of residence, smoking, and alcohol use. One-way ANOVAs were performed for marital status, educational attainment, and monthly income, as presented in **Table 3**. The results showed no significant difference in medication adherence between males and females (t = −0.310, p = 0.756). However, adherence differed significantly by education level (F = 3.531, p = 0.030), marital status (F = 8.663, p < 0.001), place of residence (t = 2.963, p = 0.003), monthly income (F = 24.462, p < 0.001), smoking (t = 3.253, p < 0.001), and alcohol use (t = −2.341, p = 0.020).

**Table 3:** Analysis of differences in medication adherence across demographic characteristics.

Variables	Items	M	SD	t/F	p	Cohen d/η^2^
Gender	Female	2.900	0.760	-0.310	0.756	0.823
	Male	2.923	0.914			
Educational background	Primary school and below	2.960^a^	0.657	3.531	0.030	0.013
	Middle school - High school	2.975^b^	0.817			
	Bachelor's degree or above	2.761^a^	0.943			
Marital status	Married	2.981^a^	0.689	8.663	<0.001	0.048
	Unmarried	2.843^b^	0.997			
	Divorced	2.921^c^	0.954			
	Widowed	2.055^a,b,c^	0.949			
Place of residence	City	2.990	0.679	2.963	0.003	0.816
	Rural area	2.771	1.007			
Monthly income level	≤3000¥	2.750^a^	0.779	24.462	<0.001	0.123
	3001-6000¥	3.029^a^	0.723			
	6001-9000¥	3.222^a^	0.752			
	9001¥ and above	2.105^a^	1.007			
Smoking	Yes.	3.008	0.695	3.253	<0.001	0.815
	No.	2.775	0.953			
Drinking alcohol	Yes.	2.829	0.909	-2.341	0.020	0.819
	No.	2.996	0.709			

M, mean; SD, standard deviation. The p-values shown in the table refer to the overall test results: independent-samples t-tests were used for variables with two categories, and one-way ANOVA was used for variables with three or more categories. For variables with three or more categories, post hoc pairwise comparisons were adjusted using the Bonferroni correction. Within each variable, identical superscript letters indicate pairwise differences that remained statistically significant after Bonferroni correction (p<0.05).

### 3.3 Descriptive statistics and correlation analysis

**Table 4** reports the means, standard deviations, skewness, and kurtosis of the study variables. Asthma patients demonstrated moderately high levels of adverse childhood experiences (M = 2.703, SD = 0.544), attachment security (M = 2.653, SD = 0.456), mentalization (M = 2.994, SD = 0.446), and medication adherence (M = 2.909, SD = 0.823), all exceeding the critical midpoint of 2.50. Skewness ranged from −0.569 to 0.254 and kurtosis from −0.224 to 1.538, meeting Kline (52) criteria for approximate normality (|skewness| ≤ 3; |kurtosis| ≤ 8).

**Table 4:** Descriptive statistics of the study variables.

Variables	M	SD	Skewness	Kurtosis
Childhood adverse experiences	2.703	0.544	0.105	0.253
Attachment security	2.653	0.456	0.254	0.446
Mentalization	2.994	0.446	-0.299	1.538
Medication adherence	2.909	0.823	-0.569	-0.224

**Figure 2:**
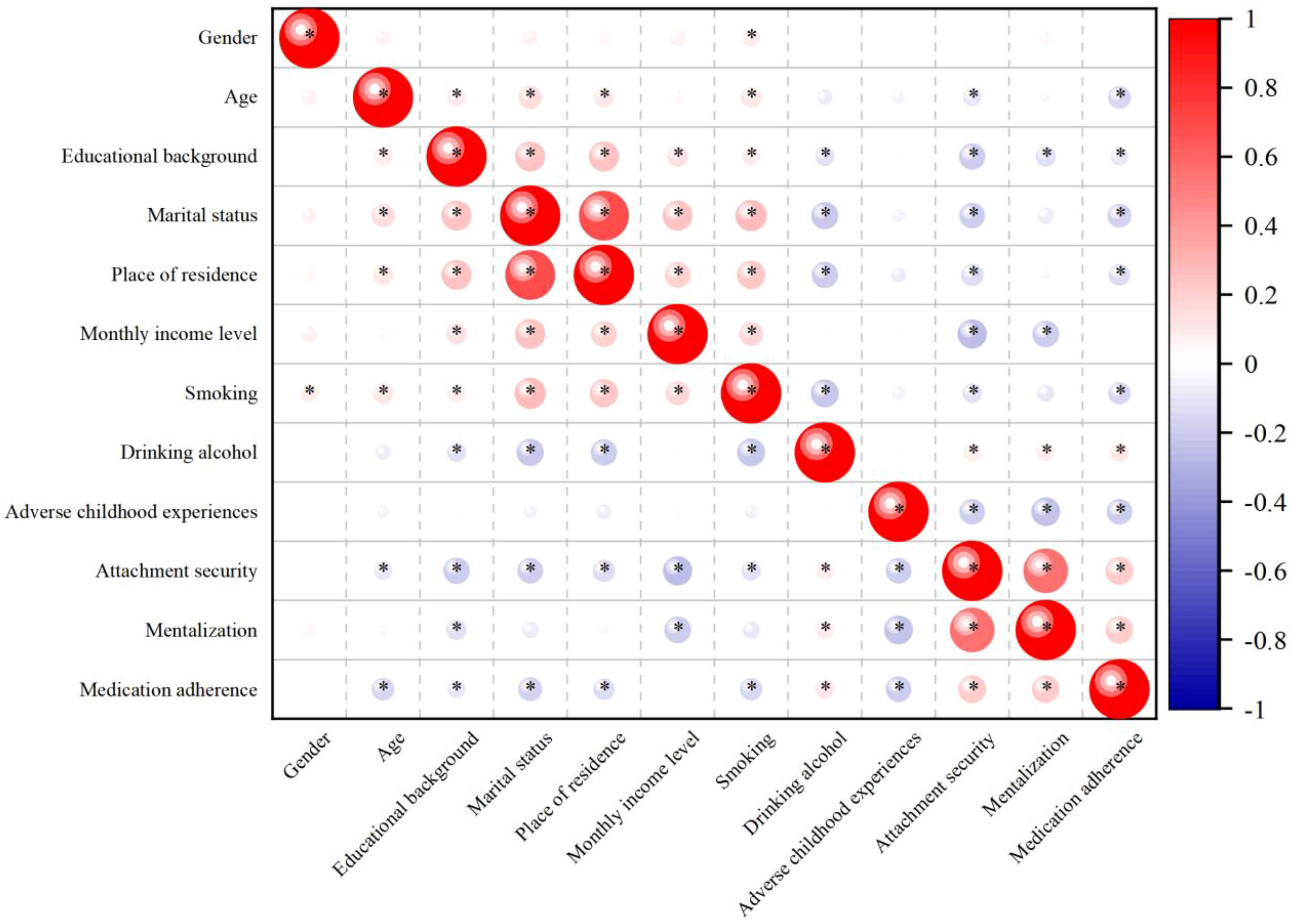
Heat map of correlations among study variables, demographic information, and health behaviors.*p < 0.05.

**Figure 2** present the correlation matrix and heatmap among study variables, demographic information, and health behaviors. Adverse childhood experiences were significantly negatively correlated with attachment security (r = −0.186, p < 0.01) and mentalization (r = −0.230, p < 0.01), indicating that individuals with greater early adversity displayed lower adult attachment security and reflective functioning. Attachment security was strongly positively correlated with mentalization (r = 0.548, p < 0.01), supporting their developmental interdependence.

Medication adherence showed a weak but significant negative association with adverse childhood experiences (r = −0.185, p < 0.01), and positive associations with both attachment security (r = 0.219, p < 0.01) and mentalization (r = 0.216, p < 0.01). These findings provide preliminary evidence for the hypothesized mediating pathways whereby early adversity influences treatment adherence indirectly through social–emotional capacities rooted in close relationships.

Age was negatively correlated with attachment security (r = −0.097, p < 0.05) and medication adherence (r = −0.144, p < 0.01). Educational level was negatively associated with attachment security (r = −0.195, p < 0.01), mentalization (r = −0.119, p < 0.01), and adherence (r = −0.093, p < 0.05). Marital status was also negatively related to attachment security (r = −0.186, p < 0.01) and adherence (r = −0.163, p < 0.01). In terms of health behaviors, smoking was negatively correlated with medication adherence (r = −0.141, p < 0.01), suggesting a co-occurrence of risk behaviors.

Overall, the correlation patterns illustrate a coherent structure in which adverse childhood experiences function as a distal risk factor, whereas attachment security and mentalization serve as potential protective factors. This structure elucidates the psychological mechanisms underlying adherence and offers a robust basis for subsequent structural modeling.

### 3.4 Chain mediation analysis of the statistical associations involving attachment security and mentalization

**Figure 3:**
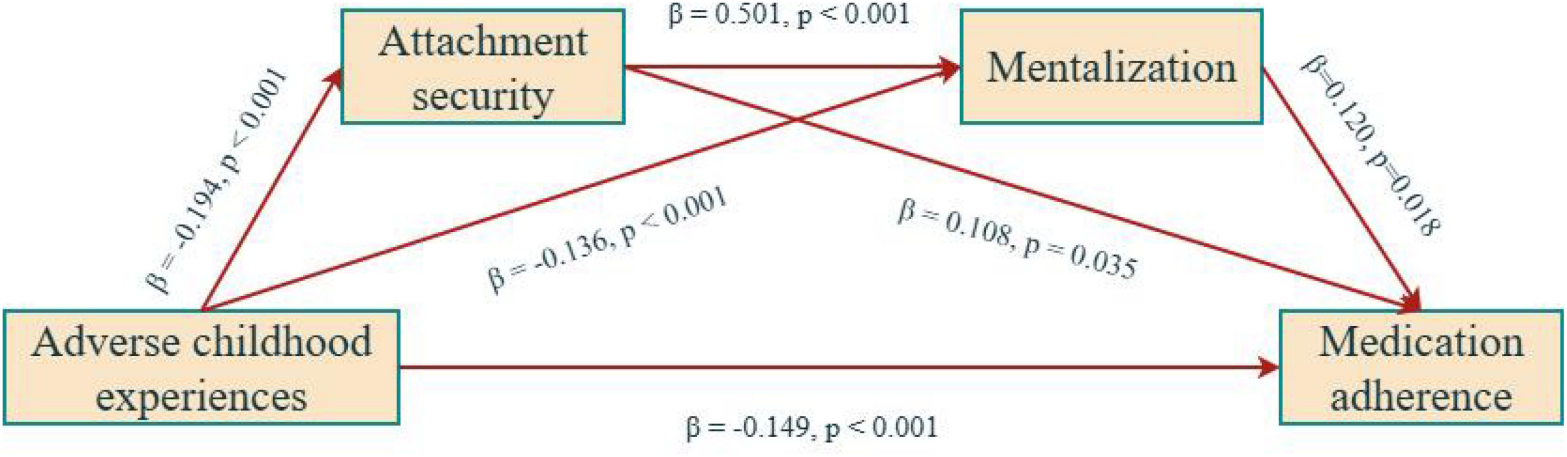
Path coefficient map of attachment security and mentalization.

Attachment security and mentalization were tested as statistical mediators in the association between adverse childhood experiences (predictor) and medication adherence (outcome), with education, marital status, residence, income, smoking, and alcohol use included as control variables (**Table 5**, **Figure 3**). The analysis employed PROCESS macro Model 6 (53). Results showed that adverse childhood experiences were significantly and negatively associated with attachment security (β = −0.194, p < 0.001), mentalization (β = −0.136, p < 0.001), and medication adherence (β = −0.149, p < 0.001). Attachment security was significantly and positively associated with mentalization (β = 0.501, p < 0.001) and adherence (β = 0.108, p = 0.035). Mentalization also was positively associated with adherence (β = 0.120, p = 0.018). Bootstrap sampling (5,000 iterations) was used to test the three mediation paths (**Table 6**).

**Table 5:** Table of multiple regression coefficients for attachment security and mentalization.

Independent variable	Model 1				Model 2				Model 3			
	Attachment security				Mentalization				Medication adherence			
	β	t	LLCI	ULCI	β	t	LLCI	ULCI	β	t	LLCI	ULCI
Adverse childhood experiences	-0.194	-4.731***	-0.231	-0.095	-0.136	-3.681***	-0.171	-0.052	-0.149	-3.467***	-0.353	-0.098
Attachment security					0.501	12.937***	0.416	0.565	0.108	2.112*	0.014	0.377
Mentalization									0.120	2.381*	0.039	0.405
R	0.3685				0.5749				0.3438			
R^2^	0.136				0.331				0.118			
F	11.603***				31.842***				7.672***			

*p < 0.05; ***p < 0.001.

**Table 6:** Decomposition of estimated direct and indirect statistical associations in the serial mediation model.

Type of effect	Effect	SE	LLCI	ULCI	Effect decomposition
Total effect	-0.300	0.064	-0.425	-0.174	100.00%
Direct effect	-0.226	0.065	-0.353	-0.098	75.22%
Indirect effect (Unstd.)	-0.074	0.023	-0.120	-0.033	24.78%
Ind 1 (Unstd.)	-0.032	0.018	-0.072	-0.001	10.61%
Ind 2 (Unstd.)	-0.025	0.013	-0.052	-0.003	8.27%
Ind 3 (Unstd.)	-0.018	0.009	-0.039	-0.002	5.90%
Indirect effect (Std.)	-0.049	0.015	-0.081	-0.022	16.33%
Ind 1 (Std.)	-0.021	0.012	-0.048	-0.0004	7.00%
Ind 2 (Std.)	-0.016	0.008	-0.034	-0.002	5.33%
Ind 3 (Std.)	-0.012	0.006	-0.025	-0.002	4.00%

Ind 1:Adverse childhood experiences → Attachment security → Medication adherence; Ind 2: Adverse childhood experiences → Mentalization → Medication adherence; Ind 3: Adverse childhood experiences → Attachment security → Mentalization →Medication adherence. UnStd. = Unstandardized estimate; Std. = Standardized estimate.

The estimated total association between adverse childhood experiences and medication adherence was −0.300, of which the estimated direct association accounted for 75.22%. Adverse childhood experiences → Attachment security → Medication adherence showed a significant partial mediation (β = −0.032, SE = 0.018, 95% CI = [−0.072, −0.001]), accounting for 10.61% of the total association. Adverse childhood experiences → Mentalization → Medication adherence also showed significant partial mediation (B = −0.025, SE = 0.013, 95% CI = [−0.052, −0.003]), accounting for 8.27%. Adverse childhood experiences → Attachment security → Mentalization → Medication adherence demonstrated a statistically significant chain mediation (B = −0.018, SE = 0.009, 95% CI = [−0.039, −0.002]), accounting for 5.90%. The indirect-to-total effect ratio for the total indirect association was 24.78%.

Regarding the specific indirect pathways, the pathway through attachment security alone was statistically detectable but small in magnitude (B = -0.032 SE = 0.018, 95% CI = [−0.072,−0.001]; β = −0.021, 95% CI = [−0.048,−0.0004]). This pathway accounted for 7.00% of the total association. Because the upper confidence bound was very close to zero and the completely standardized estimate was small, this finding should be interpreted cautiously.

The indirect pathway through mentalization alone was also statistically significant (B = −0.025, SE = 0.013; 95% CI = [−0.052,−0.003], β = −0.016, 95% CI = [−0.034,−0.002]), accounting for 5.33% of the total association. Finally, the serial pathway from adverse childhood experiences to attachment security, mentalization, and medication adherence was statistically significant (B = −0.018, SE = 0.009, 95% CI = [−0.039,−0.002], β = −0.012, 95% CI = [−0.025,−0.002], accounting for 4.00% of the total association.

Overall, these results suggest that attachment security and mentalization partially accounted for the association between adverse childhood experiences and medication adherence. However, the completely standardized estimates indicated small indirect associations.

### 3.5 Sensitivity analyses for covariate specification

To clarify the causal status of covariates, we specified a directed acyclic graph based on temporal ordering and established findings in the adverse childhood experiences literature. Because childhood adverse experiences (the exposure) occur early in life, only variables that precede the exposure can act as genuine confounders of the exposure–outcome relationship. In the present dataset, only sex and age (birth cohort) met this criterion. By contrast, educational attainment, monthly income, marital status, smoking, and alcohol use all occur in adulthood and are well-documented downstream correlates of childhood adversity; theoretically, they are better conceptualized as post-exposure variables rather than as confounders. Adjusting for variables on or downstream of the causal pathway can introduce overcontrol bias and collider-stratification bias and may attenuate or distort total and indirect effects.

**Figure 4:**
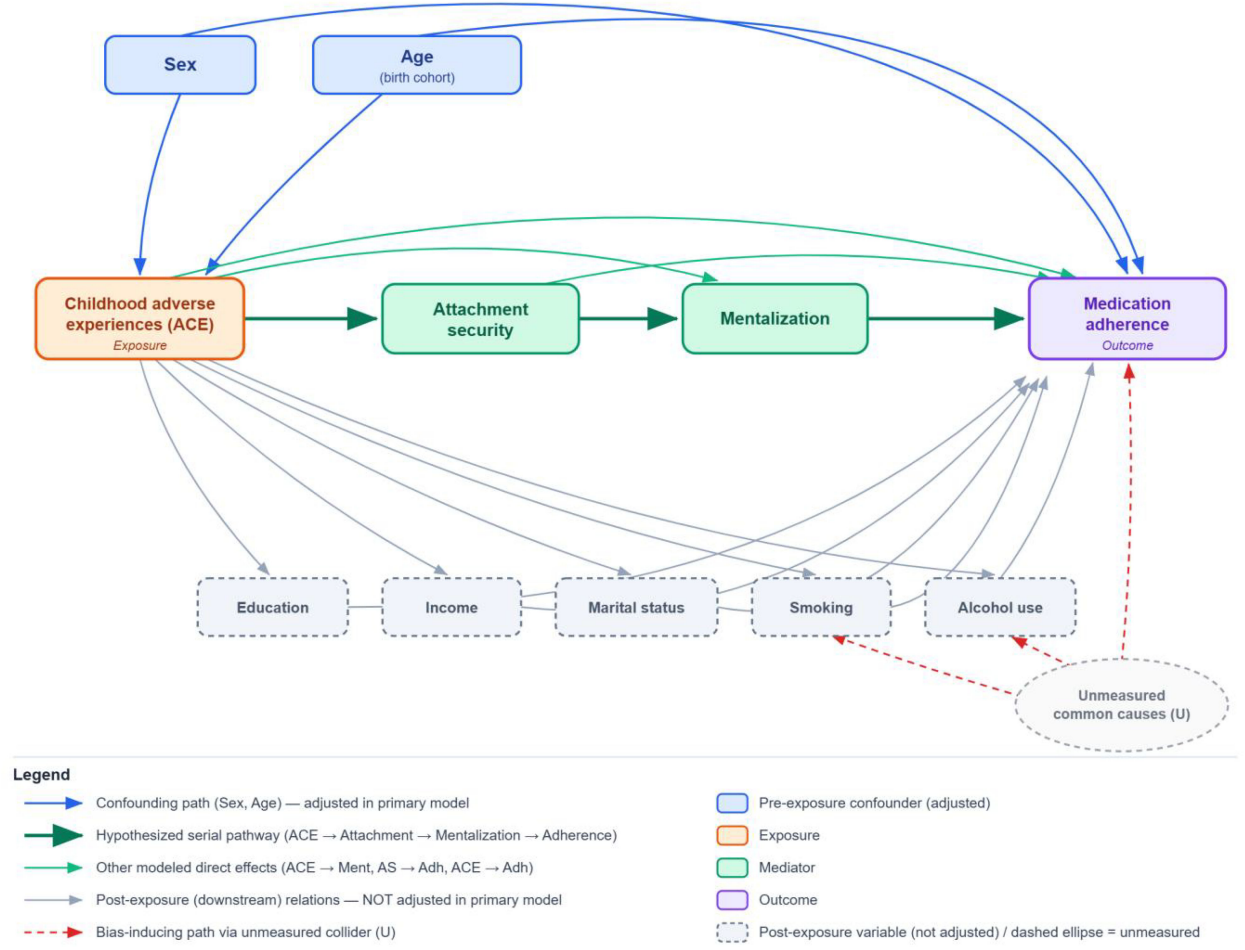
Directed acyclic graph (DAG) of the hypothesized causal structure.

The primary serial mediation model adjusted only for the pre-exposure confounders (sex and age), representing the minimally sufficient adjustment set implied by the DAG, as shown in **Figure 4**. To evaluate the robustness of the findings to covariate specification, we conducted sensitivity analyses comparing three models: (Model 1) no covariate adjustment; (Model 2, primary) adjustment for sex and age only; and (Model 3) full adjustment additionally including education, income, marital status, smoking, and alcohol use, as shown in **Table 7**. All models used the PROCESS macro Model 6 with 5,000 bootstrap resamples. We examined whether the direction, statistical significance, and magnitude of the direct, total, and three specific indirect effects were stable across specifications.

**Table 7:** Sensitivity analyses of direct and indirect effects under three covariate specifications.

Effect	Model 1: unadjusted	Model 2: sex + age (primary)	Model 3: fully adjusted
Total effect	-0.279 [-0.407,-0.152]	-0.289 [-0.416, -0.163]	-0.300 [-0.425, -0.174]
Direct effect	-0.203 [-0.332, -0.074]	-0.216 [-0.344, -0.088]	-0.226 [-0.353, -0.098]
Total indirect	-0.077 [-0.125, -0.037]	-0.074 [-0.120, -0.034]	-0.074 [-0.120, -0.033]
Ind1: ACE→AS→Adh	-0.037 [-0.081, -0.007]	-0.033 [-0.072, -0.003]	-0.032 [-0.072, -0.001]
Ind2: ACE→Ment→Adh	-0.023 [-0.050, -0.001]	-0.023 [-0.049, -0.003]	-0.025 [-0.052, -0.003]
Ind3: ACE→AS→Ment→Adh	-0.017 [-0.038, -0.001]	-0.018 [-0.038, -0.002]	-0.018 [-0.039, -0.002]

Values are completely standardized estimates with bias-corrected 95% bootstrap CIs. AS, attachment security; Ment, mentalization; Adh, medication adherence. Indicates 95% CI excluding zero.

The pattern of results was highly consistent across all three covariate specifications. The total, direct, and total indirect effects retained the same direction and statistical significance regardless of whether post-exposure variables were included. The two specific indirect pathways involving mentalization (Ind2 and the serial path Ind3) remained statistically significant in every model, whereas the attachment-security-only pathway (Ind1) was consistently small in magnitude with an upper confidence bound close to zero in all specifications, reinforcing the cautious interpretation noted earlier. Importantly, additionally adjusting for smoking, alcohol use, education, income, and marital status (Model 3) produced only trivial changes in the indirect estimates relative to the theoretically preferred minimal-adjustment model (Model 2). This indicates that our conclusions are robust to covariate specification and are not artifacts of overadjustment for post-exposure variables.

### 3.5 Latent profile analysis of attachment security and mentalization

To enhance the comprehensiveness of the research, latent profile models with 1–5 classes were constructed using Mplus 8.3. Model fit was evaluated using established criteria (54): lower AIC, BIC, and aBIC indicate better fit; entropy ≥ 0.80 indicates adequate classification; significant LMR and BLRT (p < 0.05) support improved fit over the k−1 profile model; and profile size and theoretical interpretability must be considered. Fit indices for all models are shown in **Table 8**.

**Table 8:** Model fit indices for latent profiles.

Profile	AIC	BIC	aBIC	Entropy	LMR (p)	BLRT (p)	Smallest proportion per class				
							1	2	3	4	5
1	4944.382	4987.016	4955.273	-	-	-	-				
2	4627.246	4695.461	4644.673	0.947	<0.001	<0.001	9.1%	90.9%			
3	4373.449	4467.244	4397.410	0.941	<0.001	<0.001	83.9%	8.5%	7.6%		
4	4281.690	4401.065	4312.186	0.926	0.058	<0.001	9.6%	7.3%	75.6%	7.5%	
5	4199.279	4344.234	4236.310	0.934	0.004	<0.001	2.3%	10.3%	7.0%	73.0%	7.5%

AIC, BIC, and aBIC decreased progressively with increasing profile number; however, the improvement from two to three profile was substantial, while improvements beyond three profiles diminished. The three-profile model demonstrated excellent entropy (0.94) and significant LMR (p < 0.001) and BLRT (p < 0.001) values, indicating a statistically superior fit to the two-profile model. Although four- and five-profile models also showed significant LMR and BLRT values, their additional improvements in information criteria were marginal.

The smallest class in the three-profile solution accounted for 7.6% of the sample, exceeding the 5% threshold for stability. Although four- and five-profile solutions met this criterion, their profile distinctions weakened, increasing the risk of over-extraction. The three-profile model also exhibited the highest entropy, supporting superior classification precision.

Classification accuracy was high. The average posterior probabilities for individuals assigned to Classes 1, 2, and 3 were 0.982, 0.935, and 0.924, respectively. These values exceeded the commonly used threshold of 0.70 and indicate good separation among the latent profiles. The average latent class probability matrix is presented in **Table 9**.

**Table 9:** Average latent class probabilities for most likely class membership.

Most likely class	Class 1	Class 2	Class 3
Class 1	0.982	0.010	0.009
Class 2	0.065	0.935	0.000
Class 3	0.076	0.000	0.924

Nevertheless, because the two smaller profiles accounted for relatively small proportions of the sample, and because the low attachment security–low mentalization subgroup included only approximately 39 participants, findings involving this subgroup should be interpreted cautiously and require replication in larger independent samples.

**Figure 5:**
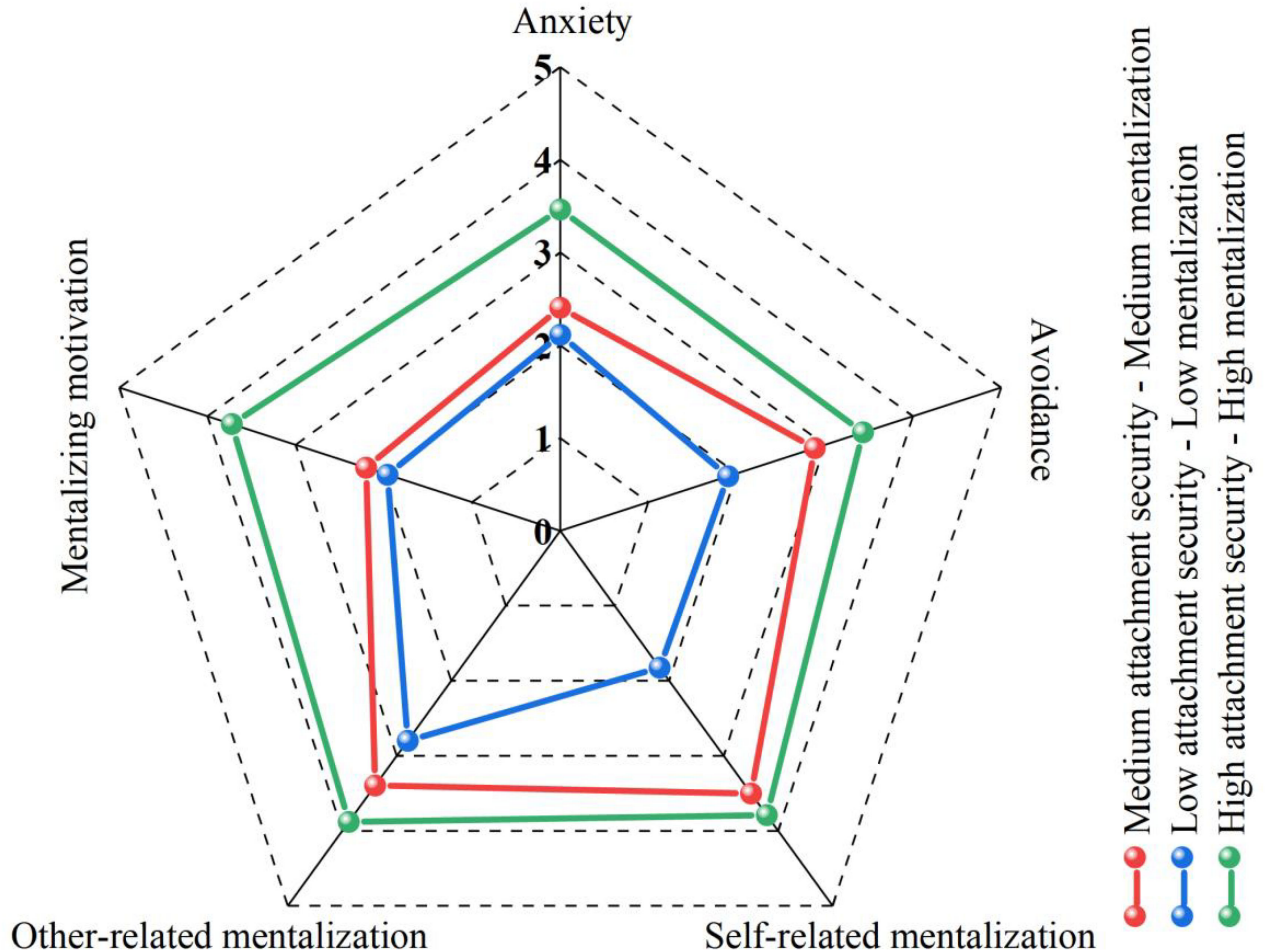
Contour plots of 3 classes of latent profile subgroups.

Given the model fit indices, classification quality, and theoretical interpretability, the three-profile model was retained. The three latent subgroups are illustrated in **Figure 5**: (1) Class 1: “Low attachment security–Low mentalization” (8.5%). Individuals in this group show marked deficits in understanding psychological states in themselves and others and tend to experience high relational insecurity, emotional dysregulation, and limited social–cognitive flexibility; (2) Class 2: “Moderate attachment security–Moderate mentalization” (83.9%). This group demonstrates generally adaptive functioning, moderate emotional regulation, and stable interpersonal behavior, representing the typical “functionally adaptive” profile. (3) Class 3: “High attachment security–High mentalization” (7.6%). These individuals exhibit strong reflective functioning, psychological resilience, and high interpersonal stability and trust.

### 3.6 Robustness test of the potential profile analysis

The final three-profile model showed adequate fit and good classification quality, with a loglikelihood of -2164.724, AIC of 4373.449, BIC of 4467.244, sample-size adjusted BIC of 4397.410, and entropy of 0.941. The estimated profile proportions were 83.92%, 8.48%, and 7.60%, respectively. In the bootstrap robustness analysis, all 500 requested bootstrap draws were successfully completed, and the best loglikelihood value was replicated. These findings support the robustness of the final three-profile solution, as shown **Table 10**.

**Table 10:** Bias-corrected bootstrap confidence intervals.

Class	Variable	Estimate	95% CI
Class 2	Anxiety	2.404	2.357–2.460
	Avoidance	2.887	2.840–2.939
	Self-related mentalization	3.501	3.448–3.548
	Other-related mentalization	3.395	3.338–3.447
	Mentalizing motivation	2.201	2.159–2.255
Class 1	Anxiety	2.113	1.832–2.312
	Avoidance	1.906	1.733–2.103
	Self-related mentalization	1.826	1.532–2.101
	Other-related mentalization	2.798	2.446–3.066
	Mentalizing motivation	1.958	1.800–2.125
Class 3	Anxiety	3.464	3.216–3.684
	Avoidance	3.434	3.273–3.565
	Self-related mentalization	3.791	3.622–3.937
	Other-related mentalization	3.879	3.754–4.047
	Mentalizing motivation	3.724	3.502–3.917

### 3.7 One-way ANOVA of latent subgroups and medication adherence

To assess whether the latent subgroups differed in medication adherence, a one-way ANOVA was conducted with the three latent profiles as the independent variable and adherence as the dependent variable. Medication adherence in the “High attachment security–High mentalization” subgroup (M = 3.212, SD = 0.824) was significantly higher than in the “Moderate attachment security–Moderate mentalization” group (M = 2.934, SD = 0.802) and the “Low attachment security–Low mentalization” group (M = 2.381, SD = 0.830). Thus, the latent profiles differed significantly in medication adherence (F(2, 522) = 12.217, p < 0.001, η² = 0.045).

**Table 11** presents the comparison of demographic characteristics across the three latent classes. Significant differences were observed in educational background (F = 11.661, p = 0.020), monthly income level (F = 18.707,p = 0.005), and drinking alcohol (χ^2^ = 9.560,p=0.008). No significant differences were found across the three classes in gender, marital status, place of residence, smoking, or age.

**Table 11:** Demographic characteristics of participants by latent class membership.

Variables	Items	Class 1	Class 2	Class 3	χ²/F	p
Gender	Female	272	29	20	2.327	0.312
	Male	171	14	19		
Educational background	Primary school and below	114	7	17	11.661	0.020
	Middle school - High school	204	17	13		
	High school and above	125	19	9		
Marital status	Married	285	22	29	9.785	0.134
	Unmarried	95	14	8		
	Divorced	48	4	0		
	Widowed	15	3	2		
Place of residence	City	277	26	27	0.805	0.669
	Rural area	166	17	12		
Monthly income level	≤3000¥	141	13	23	18.707	0.005
	3001-6000¥	170	12	11		
	6001-9000¥	101	11	4		
	9001¥ and above	31	7	1		
Smoking	Yes.	259	21	21	1.695	0.429
	No.	184	22	18		
Drinking alcohol	Yes.	239	23	11	9.56	0.008
	No.	204	20	28		
Age	M ± SD	54.20 ± 16.699	53.47 ± 17.401	50.51 ± 16.061	0.888	0.412

Values are presented as n for categorical variables and mean ± standard deviation for age. Pearson’s chi-square tests were used for categorical variables, and one-way ANOVA was used for age. The χ^2^/F column reports χ^2^ values for categorical variables and F values for age.

## 4 Discussion

### 4.1 Variable-centered analysis

Drawing on a variable-centered analytical framework, this study systematically examined the statistical associations linking adverse childhood experiences influence medication adherence among adults with asthma. It also provides empirical evidence from a Chinese adult asthma sample supporting a statistically chained mediation pathway involving attachment security and mentalization. Specifically, higher levels of childhood adverse experiences were associated with lower medication adherence both directly and indirectly through lower attachment security and lower mentalization. This pattern is highly consistent with developmental psychopathology models emphasizing continuity across the sequence of early adversity → relational capacity → self-regulation → health behavior, reinforcing the cross-stage and cross-system impacts of early psychosocial risks. Within the context of chronic disease management, our findings underscore that childhood adversity may persistently shape adult health decision-making, suggesting that clinical asthma management should move beyond traditional biomedical approaches to incorporate psychosocial mechanisms. Importantly, however, the completely standardized indirect effects were small. Thus, the findings should be interpreted as indicating statistically detectable psychological pathways rather than large or clinically decisive mediation effects.

Regarding the statistically mediating role of attachment security, the study identifies a unique and significant partial mediation effect, suggesting that early relational insecurity reduces trust in others and undermines emotional regulatory resources. Individuals with insecure attachment may be more susceptible to avoidance, doubt, or inconsistent medication use when facing long-term structured asthma treatment regimens. These findings align with prior research indicating that insecurely attached individuals often show poorer adherence and diminished capacity to follow medical advice in chronic illness contexts (25). Notably, our sample was drawn from a Chinese cultural context, where social-emotional dependence, interpersonal trust, and family structures may amplify the influence of attachment on health behavior, underscoring its unique importance in East Asian populations.

Mentalization, the second key mediator, also showed a significantly association with medication adherence and contributed independently to the pathway linking adverse childhood experiences to treatment persistence. Individuals with low mentalization often struggle to accurately interpret the relationship between their symptoms and emotions and may engage in maladaptive health behaviors in anxiety- or stress-provoking situations (55), such as overreliance on rescue medication or neglect of maintenance therapy. This study provides the first empirical evidence supporting a mentalization-to-asthma-behavior-management pathway. More importantly, the validated chained mediation—where attachment security enhances mentalization, which in turn improves adherence—highlights the joint role of emotional regulation and reflective functioning in long-term asthma management and strengthens theoretical models addressing psychological mechanisms in chronic disease care.

### 4.2 Person-centered analysis

Using latent profile analysis, this study identified three distinct psychological subgroups characterized by differences in attachment security and mentalization: “High attachment–High mentalization,” “Moderate attachment–Moderate mentalization,” and “Low attachment–Low mentalization.” These findings reveal notable psychological heterogeneity among asthma patients and suggest that asthma management should not be conceptualized as a homogeneous behavioral phenomenon. Rather, risk stratification should be informed by underlying psychological structures.

The “Low attachment–Low mentalization” group, though accounting for only 8.5% of the sample, displayed marked psychological vulnerability. This subgroup is consistent with profiles described in prior literature as “avoidant–anxious combined” or “vulnerable mentalization-impaired,” and may demonstrate unstable adherence, emotionally driven symptom interpretation, or basic treatment mistrust. However, the stability, prevalence, and clinical significance of this subgroup need to be confirmed in larger, more diverse, and longitudinal samples. Clinically, this subgroup may be considered a candidate group for further assessment and potentially more intensive psychosocial support, but not as a basis for broad generalization or categorical risk labeling.

The “Moderate attachment–Moderate mentalization” subgroup, comprising 83.9% of the sample, represents the predominant psychological pattern among asthma patients. Their functioning is neither severely impaired nor highly optimal; rather, their behavioral stability is best described as “moderately consistent.” This supports the “moderate adaptation model” in health behavior research, suggesting that most patients occupy a transitional state in which adherence is influenced by external support, illness perceptions, and treatment experience. Behavioral reinforcement and continuous monitoring are particularly important for this group.

In contrast, the “High attachment–High mentalization” subgroup (7.6%) demonstrated the highest adherence, indicating that strong relational security and robust reflective functioning jointly facilitate stable and adaptive health behaviors. These individuals tend to trust treatment recommendations, show better insight into bodily sensations, and maintain structured health routines even under stress. Identifying this subgroup is valuable for understanding psychological resilience in asthma management and contributes to emerging research on resilience-based pathways. To our knowledge, this study provides the first evidence from a Chinese asthma sample supporting a three-profile psychological structure, offering direct theoretical implications for precision medicine and personalized asthma management.

### 4.3 Cultural specificity and comparison with western studies

The findings also need to be understood within the Chinese cultural context and in comparison with Western studies. Consistent with Western research, our results suggest that childhood adversity may undermine adult health behavior through reduced relational security, poorer emotion regulation, and impaired reflective functioning. This supports the possibility that the pathway from early adversity to treatment adherence through attachment and mentalization reflects a partially cross-cultural psychological mechanism. However, Chinese cultural characteristics may shape the salience and expression of this pathway. In China, family interdependence, filial responsibility, and relational harmony remain central to illness management; family members often provide treatment reminders, emotional support, financial assistance, and decision-making input. Therefore, childhood adversity within the family may have particularly enduring effects by weakening later relational trust and perceived availability of support. In contrast, Western adherence research often places greater emphasis on individual autonomy, self-management, and shared decision-making.

Moreover, cultural norms of emotional restraint and indirect expression in East Asian societies may make it more difficult for some patients to identify and verbalize emotional distress, thereby increasing the risk that anxiety or distress is confused with respiratory symptoms. This may partly explain the role of mentalization in asthma medication adherence. Respect for medical authority and family monitoring may also buffer adherence problems among some Chinese patients, whereas stigma toward psychological distress may inhibit open discussion of non-adherence or emotional difficulties. Thus, the present findings extend Western attachment- and mentalization-based models by demonstrating their relevance in a Chinese asthma sample while highlighting the importance of culturally sensitive, family-informed, and trauma-informed adherence interventions.

### 4.4 Practical implications

This study offers several practical implications for asthma management. First, variable-centered results highlight the non-negligible impact of adverse childhood experiences, suggesting the need to shift from a disease-centered to a trauma-informed integrated management model. Incorporating early adversity screening into clinical assessments may help clinicians identify psychological barriers underlying poor adherence and develop more targeted interventions. For individuals with insecure attachment or impaired mentalization, medication education alone is often insufficient; trauma-informed and relationship-focused psychological interventions are essential.

Second, person-centered findings reveal substantial psychological heterogeneity and support stratified management approaches. Patients in the “Low attachment–Low mentalization” group should be prioritized for intensive psychological interventions, such as attachment-based therapy, mentalization-based treatment, or emotion regulation training, to foster more structured treatment behaviors. The “Moderate attachment–Moderate mentalization” group would benefit from behavioral incentives, health education, and emotion management, while the “High attachment–High mentalization” group requires only minimal follow-up to maintain stability, optimizing resource allocation.

Finally, the study highlights the need to integrate psychological mechanisms into asthma care. Attachment security and mentalization—stable psychosocial traits—can serve as predictors of long-term adherence. Based on the chained mediation model, healthcare institutions could develop rapid screening tools to identify adherence risks and design integrated management systems involving multidisciplinary collaboration across respiratory medicine, psychology, and nursing. Such an approach may reduce exacerbation risk, decrease resource waste, and enhance the long-term sustainability of chronic disease management.

### 4.5 Limitations and future directions

Despite its methodological strengths, this study has several important limitations that should be considered when interpreting the findings. First, the cross-sectional design prevents firm conclusions regarding causal directionality. Although the hypothesized ordering of childhood adverse experiences, attachment security, mentalization, and medication adherence was grounded in developmental psychopathology, attachment theory, and mentalization theory, all variables were assessed at a single time point. Therefore, temporal precedence among attachment security, mentalization, and adherence could not be empirically established. The mediation results should be interpreted as theoretically informed indirect statistical associations rather than evidence of causal mediation. Alternative explanations are possible. For example, poor asthma control or low medication adherence may increase emotional distress, which could influence current perceptions of attachment security or self-reflective functioning. Longitudinal studies with repeated assessments are needed to examine whether childhood adversity prospectively predicts changes in attachment security, mentalization, and medication adherence over time.

Second, the retrospective self-report assessment of childhood adverse experiences may be affected by recall bias and social desirability bias. Childhood adversity involves sensitive family-related experiences, including neglect, abuse, and household dysfunction. Participants may underreport such experiences because of shame, privacy concerns, filial norms, or the desire to present themselves and their families in a socially acceptable manner. This issue may be particularly salient in the Chinese cultural context, where family reputation, relational harmony, and filial responsibility are strongly emphasized. Such underreporting may have led to an underestimation of childhood adversity and may have attenuated the observed associations. Conversely, participants currently experiencing psychological distress, poor asthma control, or dissatisfaction with treatment may recall childhood experiences more negatively, potentially inflating associations among self-reported variables. Therefore, the present findings should be interpreted as associations based on subjective retrospective reports rather than objective records of early-life adversity.

Third, medication adherence was assessed using a self-report scale only. Although the instrument used in this study has demonstrated acceptable reliability and validity in chronic disease populations, self-reported adherence may be influenced by recall errors, misunderstanding of prescribed regimens, and social desirability. Patients may overreport adherence because they wish to satisfy perceived expectations from clinicians or researchers. Future studies should combine self-report measures with objective or semi-objective adherence indicators, such as electronic inhaler monitoring, pharmacy refill data, prescription claims, dose counters, or clinician-rated adherence. Such multimethod assessment would reduce measurement bias and provide a more comprehensive picture of adherence behavior in asthma management.

Fourth, participants were recruited exclusively from tertiary hospitals in Beijing, which may limit the generalizability of the findings. Patients treated in tertiary hospitals may differ from those in community hospitals, primary care settings, rural areas, or less-resourced regions in terms of disease severity, healthcare access, socioeconomic status, health literacy, and exposure to specialized asthma education. Beijing is also a highly urbanized region with relatively concentrated medical resources. Therefore, the present sample may not fully represent the broader population of adults with asthma in China. Although the sample included both urban and rural residents and participants with different income and education levels, selection bias cannot be excluded. Future studies should recruit larger and more geographically diverse samples from multiple provinces, community clinics, primary care institutions, and rural healthcare settings to test the robustness and external validity of the current findings.

Fifth, although the three-profile solution in the latent profile analysis showed good entropy, high posterior classification probabilities, and bootstrap-supported stability, the two smaller latent profiles accounted for relatively small proportions of the sample. In particular, the low attachment security–low mentalization subgroup included only a limited number of participants. Small latent classes may be more sensitive to sampling variation and may not replicate consistently across independent datasets. Therefore, these profiles should be regarded as preliminary psychological risk configurations rather than definitive clinical categories. Future research should validate the profile structure in larger, multicenter, and longitudinal samples and examine whether these profiles prospectively predict objective adherence and asthma outcomes.

Sixth, although the indirect pathways through attachment security and mentalization were statistically significant, their completely standardized effect sizes were small, and the confidence interval for the attachment-security-only pathway approached zero. These findings suggest that attachment security and mentalization may represent modest psychological pathways rather than dominant determinants of medication adherence. Asthma adherence is likely shaped by multiple biomedical, behavioral, interpersonal, and structural factors, including symptom burden, inhaler technique, medication cost, treatment beliefs, patient–provider communication, family support, depression, anxiety, and healthcare accessibility. Future studies should integrate these factors into multilevel or network models to clarify how psychological mechanisms interact with clinical and contextual determinants of adherence.

Finally, because this study was observational, residual confounding cannot be ruled out. Although sensitivity analyses using different covariate specifications yielded consistent findings, unmeasured variables such as asthma severity, duration of illness, comorbid anxiety or depression, perceived treatment burden, family support, and patient–provider relationship quality may have influenced both psychological functioning and adherence. Future research should incorporate more comprehensive clinical indicators and psychosocial covariates, and randomized or quasi-experimental intervention studies are needed to determine whether improving attachment-related security or mentalization capacity can lead to meaningful improvements in medication adherence and asthma outcomes.

## 5 Conclusion

This study offers three main take-home messages. First, adverse childhood experiences were associated with poorer medication adherence among adult asthma patients. Second, attachment security and mentalization may represent important psychological pathways through which early adversity relates to adherence behavior. Third, asthma patients showed heterogeneous psychological profiles, but smaller latent profiles should be interpreted as preliminary and require validation in larger and longitudinal samples. These findings suggest that attachment- and mentalization-informed perspectives may enrich psychosocial assessment and adherence-support strategies in asthma care, while future prospective research is needed to clarify causality and clinical utility.

## Data Availability

The raw data supporting the conclusions of this article will be made available by the authors, without undue reservation.
